# Co-chaperones DNAJA1 and DNAJB6 are critical for regulation of polyglutamine aggregation

**DOI:** 10.1038/s41598-020-65046-5

**Published:** 2020-05-18

**Authors:** Claudio Rodríguez-González, Shiying Lin, Sertan Arkan, Christian Hansen

**Affiliations:** Molecular Neurobiology, Department of Experimental Medical Science, Lund University, BMC B11, 22184 Lund, Sweden

**Keywords:** Mechanisms of disease, Mechanisms of disease, Protein folding, Protein folding

## Abstract

Huntington’s disease (HD) is caused by CAG repeat expansion in the huntingtin gene. The expanded polyglutamine (polyQ) repeat of the encoded protein leads to protein misfolding and aggregation, resulting in increased neuronal cell death. DNAJ co-chaperones play a crucial role in transferring misfolded/unfolded proteins to HSP70 chaperones, which play an essential role for protein folding. Here, we investigated the effect of knock out (KO) of three individual DNAJ genes in HEK293 cells expressing polyglutamine74exon1 huntingtin (polyQ74htt). Flourescence microscopy analysis revealed that KO of DNAJB6 resulted in a 5-fold increase in polyQ74htt aggregation and that DNAJA1 KO resulted in a 4-fold decrease of polyQ74htt aggregation. KO of DNAJB1 did not change the propensity of polyQ74htt to aggregate in cells. These findings where confirmed both by fluorescence microscopy analysis and filter trap assay (FTA). DNAJB6 KO cells displayed an increased rate of cell death as assessed by trypan blue exclusion and propidium iodide (PI) uptake assays. These results demonstrate that the DNAJ proteins DNAJA1 and DNAJB6 can modulate polyQ aggregation in opposite manners, and thus that fine-tuning the cellular levels of DNAJ proteins is critical for suppression of polyQ aggregation and cell survival.

## Introduction

PolyQ diseases are characterized by expanded regions of CAG repeats, encoding polyQ stretches at the N-terminus of the proteins^1^. One of these diseases is Huntington’s disease (HD), which is characterized by neurodegeneration in the cerebral cortex and basal ganglia, that causes a progressive loss of motor control, as well as cognitive symptoms^2^. The length of the polyQ expansion that cause disease progression varies with the gene targeted. In HD, an expansion of at least 40 CAG triplets in one allele of the protein Huntingtin is enough to trigger the disease^3^. Unfortunately, so far there is no available treatment for HD, only symptomatic medication that does not stop the progression of the disease^4^.

The 70 kDa heat shock proteins (HSP70s) play an important role in the maintenance of protein homeostasis in cells^5^. HSP70s are involved in a range of mechanisms, such folding of newly synthesised proteins, refolding of misfolded proteins and targeting of proteins for degradation^6^. However, the HSP70 chaperones need the assistance of co-chaperones to carry out their function. One major group of such co-chaperones is the DNAJ family of proteins, which consists of more than 40 different members^7^. These proteins can recruit misfolded or unfolded proteins and transfer them to the HSP70 proteins in an ATP dependent manner^8^. Therefore, the DNAJ proteins are important for regulation of protein homeostasis. Indeed, it has been shown that several DNAJ genes have been linked to diseases that involve accumulation of misfolded proteins, which includes neurodegenerative diseases, in which aggregates of amyloid proteins are formed^9–11^.

Interestingly, previous work by Kampinga lab and others have suggested that some DNAJ proteins, in particular DNAJB6 and DNAJB8 may be important for suppressing polyQ protein aggregation^12,13^. However, these studies were restricted to evaluation by DNAJ protein overexpression^12,13^ and furthermore endogenous expression of DNAJB8 is largely restricted to testis^14^. In this study, we investigated if Crispr/CAS9 mediated gene knock out (KO) of 3 selected DNAJ genes, that had all prior been linked to amyloid protein aggregation, DNAJB6^15,16^, DNAJB1^17^ and DNAJA1^18^ would modulate polyQ74htt aggregation. Here, we demonstrate that two of these genes, DNAJB6 and DNAJA1, affect polyQ aggregation with opposing effects and that increased aggregation seen upon KO of DNAJB6 resulted in increased cell death.

## Materials and Methods

### Cell Culture, Transfection, plasmids and genome editing

Stable expression of green fluorescent protein (GFP) fused with 74 polyQ repeat (PolyQ74) and exon 1 of the huntingtin encoding gene in human embryonic kidney 293 (HEK293) cells were generated, using 200 µg/ml G418 selection marker and transfection with polyQ74htt expression plasmid (addgene# 40262). These cells were maintained in DMEM with GlutaMAX-I (Thermo Fisher Scientific) supplemented with 10% FBS (Thermo Fisher Scientific) and 1% penicillin/streptomycin (Thermo Fisher Scientific).

CRISPR/Cas9 targeting sequences for DNAJA1, DNAJB1 and DNAJB6 genes were designed using the Sanger program: http://www.sanger.ac.uk/htgt/wge/find_CRISPRs. The sequences used to generate DNAJA1 KO with 2 different guides were 5′caccgcaacttactacgatgttttg3’, 5′aaaccaaaacatcgtagtaagttgc3′, 5′caccgggtcaaacccaatgctactc 3′ and 5′aaacgagtagcattgggtttgaccc3′: the sequences used to generate DNAJB1 KO with 2 different guides were 5′caccgtctcctcgtccgacgcgccg3′, 5′aaaccggcgcgtcggacgaggaga3′, 5′caccgcgggtcgctgagcacgtcgt3′ and 5′aaacacgacgtgctcagcgacccgc3′: the sequences used to direct DNAJB6 KO 5′caccgttacgcctttttaatatcct 3′, 5′aaacaggatattaaaaaggcgtaac3′, 5′caccgggaggcatatgaagtgctgt3′ and 5′aaacacagcacttcatatgcctccc′3. Cloning into Cas9-Cherry construct (Derived from Cas9-GFP construct, Addgene: cat# 48138) and transformation of (HEK293)-polyQ74 cells were done according to protocols described in^19^. Cherry-Cas9 positive cells were single cell sorted into 96 well plates after 36 hours of incubation and single cell clones were analyzed for Cas9 induced KO’s, by western blot analysis.

For re-introduction of DNAJB6 or DNAJA1 into cells we expressed Cherry-DNAJB6b or Cherry co-expressed with DNAJA1. cDNA’s encoding human V5 tagged DNAJB6b and DNAJA1 in pcDNA3 vectors were kind gifts from Harm Kampinga’s lab (University of Groningen, Holland). With regard to the DNAJB6 cDNA, we inserted the cDNA into a pACGFP-C1 vector (clontech) in which Cherry had replaced GFP. For insertion of DNAJB6 into this vector, we amplified DNAJB6 by PCR and inserted the cDNA by use of flanking primers containing restriction enzyme overhangs for XhoI and BamHI (5′ tcgaggatccctagtgattgcctttggtcgacttcttc 3′ and 5′ gtcaggatccttacttgttatccaagcgcagcagctg 3′).

### SDS-PAGE Western Blotting, antibodies and reagents

Cells were lysed in lysis buffer (0.5% Triton X-100, 50 nM Tris HCl, 175 mM NaCl, and 5 mM EDTA, pH 8, 1:100 protease inhibitor cocktail (Sigma-Aldrich; P8340) and cell lysates were incubated on ice for 20 minutes, the debris was spun down at 10,000 g for 10 minutes at 4 °C, the supernatants were collected and Laemmli buffer + 10% 1 M DTT was added. 15 µg of protein was loaded onto 10% or 12% SDS-PAGE polyacrylamide gels for electrophoresis and then transferred to PVDF membranes by use of a Trans-Blot Turbo Transfer System (Bio-Rad). The membranes were blocked in 5% (w/v) skim milk in PBS containing 0.05% Tween (PBS-T) for one hour. Primary antibodies used were anti-DNAJA1 (1:2000, Protein tech biosite; Cat#11713-1-AP), anti-DNAJB6 (1:2000, Abcam; Cat#ab198995), anti-HSP40/DNAJB1 (1:2000, Enzo Life Sciences; Cat#ADI-SPA-400-D), anti-β-actin (1:10000, Sigma-Aldrich; Cat#A3854). All incubations with antibodies were performed in 2–3% milk in PBS-T. Incubation of blots with primary antibodies was performed at 4 °C overnight, followed by three x 5 minutes washes with PBS-T and finally incubation with anti-rabbit HRP conjugated secondary antibody (1:5000, Dako cat# P0448) for one hour at room temperature. After washes in PBS-T, the membranes were analyzed using ImmunoCruz Western Blotting Luminol Reagent (Santa Cruz Biotechnology). Luminescence signals from membranes were imaged by the use of the ChemiDoc XRS + system (Bio-Rad).

In insoluble fraction western blot experiment, cell were lysed in NP40 lysis buffer (0.5% NP40, 50 mM Tris HCl, 175 mM NaCl, and 5 mM EDTA, pH 8, 1:100 protease inhibitor cocktail (Sigma-Aldrich; P8340)), and cell lysates were incubated on ice for 20 minutes, the cell pellet was spun down at 15,000 g for 10 minutes at 4 °C, the supernatants were collected. The pellets were resuspended with 5% NP40 lysis buffer + Laemmli buffer + 10% 1 M DTT. 20 µg of protein was loaded onto 10% SDS-PAGE polyacrylamide gels for electrophoresis and then transferred to PVDF membranes by use of a Trans-Blot Turbo Transfer System (Bio-Rad). The membranes were blocked in 5% (w/v) skim milk in PBS containing 0.05% Tween (PBS-T) for one hour. Primary antibodies used were anti-GFP (1:1000, ThermoFisher: MA5-15256) anti-β-actin (1:10000, Sigma-Aldrich; Cat#A3854). All incubations with antibodies were performed in 2–3% milk in PBS-T. Incubation of blots with primary antibodies was performed at 4 °C overnight, followed by three x 5 minutes washes with PBS-T and finally incubation with anti-mouse HRP conjugated secondary antibody (1:5000, Dako) for one hour at room temperature. After washes in PBS-T, the membranes were analyzed using ImmunoCruz Western Blotting Luminol Reagent (Santa Cruz Biotechnology). Luminescence signals from membranes were imaged by use of the ChemiDoc XRS + system (Bio-Rad).

### Fluorescence microscopy and cell line comparison

Glass coverslip in each well of 24-well plate was coated for 20 minutes with 0.1 mg/ml poly-L-lysine. About 80,000 cells per well were seeded and incubated overnight. Cells were rinsed with PBS and fixed in 4% Paraformaldehyde (PFA) for 20 minutes. Then cells were washed 3 times with 500 µl PBS. The coverslips were mounted onto glass slides with Vectashield mounting medium containing DAPI (Vector Laboratories). For polyQ-74 aggregates quantification in the cell line, epifluorescence microscopy for imaging and counting aggregates was done by using Nikon eclipse 80i microscope at 20x-60X magnification. For each independent experiment, at least 200 cells were counted and the percentage of cells that contained aggregates (GFP positive puncta) were counted. The experiments were not randomized nor blinded.

### Cell apoptosis with trypan blue exclusion and PI tests

#### Trypan blue

For each DnaJKO cell line, 80.000 cells per well were seeded overnight, in 5 independent experiments. Cells were trypsinized, spun down at 400 g for 1 minute and resuspended in 100 µL of medium. Subsequently, 10 µl of cell suspension was mixed with 10 µl 0.4% trypan blue dye (Bio-Rad; 450021) and 10 µl of this cell/trypan blue suspension was added into a cell counting slide (Bio-Rad). Cell viability was determined by the result analyzed by a TC20 automated cell counter (Bio-Rad).

#### PI

Cells were harvested, resuspended in cell culture media with 2 µl/ml of PI (50 mg/ml (BD Pharmingen™) and immediately analyzed on a flow cytometer (FACS Aria III, BD FACS Diva software 8.0.1, BD Biosciences). PI was excited at 561 nm and detected using a 610/20 bandpass filter. The fraction of dead cells was measured as PI + cells out of single cells.

### Filter trap assay

Filter Trap Assay (FTA) was done according to the protocol described by Waarde-Verhagen and Kampinga^20^. Briefly: Protein samples were prepared by seeding 1.000.000 cells in a 6-well plate and left for incubation overnight. The cells were then washed with 1 ml PBS and subsequently, 200 µl lysis buffer was added (FTA buffer: (pH 8.0: 10 mM Tris-Cl and 150 mM NaCl) + 2% SDS). The cells were scraped into Eppendorf vials and the lysates were then sonicated for 5 seconds at 50 W (33% amplitude) with a microtip sonifier. Protein concentrations were measured and samples were then brought to 40 µg in 400 µl of lysis buffer. 1 M DTT was added to a final concentration of 50 mM to the stock samples, followed by heating of the samples to 96 °C for 5 minutes. 1:5 to 1:25 serial dilutions were made from boiled stock samples, to achieve a final volume of 400 µl. Samples with 100 µg protein extract were loaded in slots and filtered through a 0.22 µm Cellulose Acetate membrane (Sterlitech Corporation; Cat#CA022005), which had been pre-equilibrated  using wash buffer (FTA buffer+0.1% SDS) . The slots were washed three times with 100 µl wash buffer and following the blotting apparatus was disassembled, the membrane was taken out and washed twice with 0.1% PBS-T. The membrane was blocked with 5% skim milk powder in 0.1% PBS-T at room temperature for 1 hour and incubated overnight at 4 °C with mouse GFP primary antibody (1:5000 in 0.1% PBS-T with 3% BSA, Thermo Fischer Scientific; Cat#MA5-15256). The next day, the membrane was washed with 0.1% PBS-T and then incubated with goat anti-mouse IgG HRP-conjugated secondary antibody (1:5000) DAKO cat#P0447) for 1 hour at room temperature. After three x 5 min washes with PBS-T, enhanced chemiluminescence (ECL) substrate was applied to detect polyQ protein using ImmunoCruz Western Blotting Luminol Reagent (Santa Cruz Biotechnology; Cat#sc-2048). The results were visualized using a ChemiDoc XRS + system (Bio-Rad). Accurate quantification of the bands was accomplished by Image J software (National Institutes of Health).

### Statistical analysis

Quantitative data analysis was done by using GraphPad Prism (version 6, US). Data are expressed as mean ± SE based on three independent experiments or more, in all cases. Statistical significance was calculated using one-way analysis of variance (ANOVA) assuming normal distribution, followed by post-hoc Tukey test where ^∗^P ≤ 0.05, ^∗∗^P ≤ 0.01 and ^∗∗∗^P ≤ 0.001. Additional, statistical analysis using Mann-Whitney non-parametric test of all the data analyzed by one-way analysis of variance (ANOVA) assuming normal distribution, showed significant difference ^∗^P ≤ 0.05, for all data in which we detected significant differences using one-way ANOVA.

## Results

**Figure 1:**
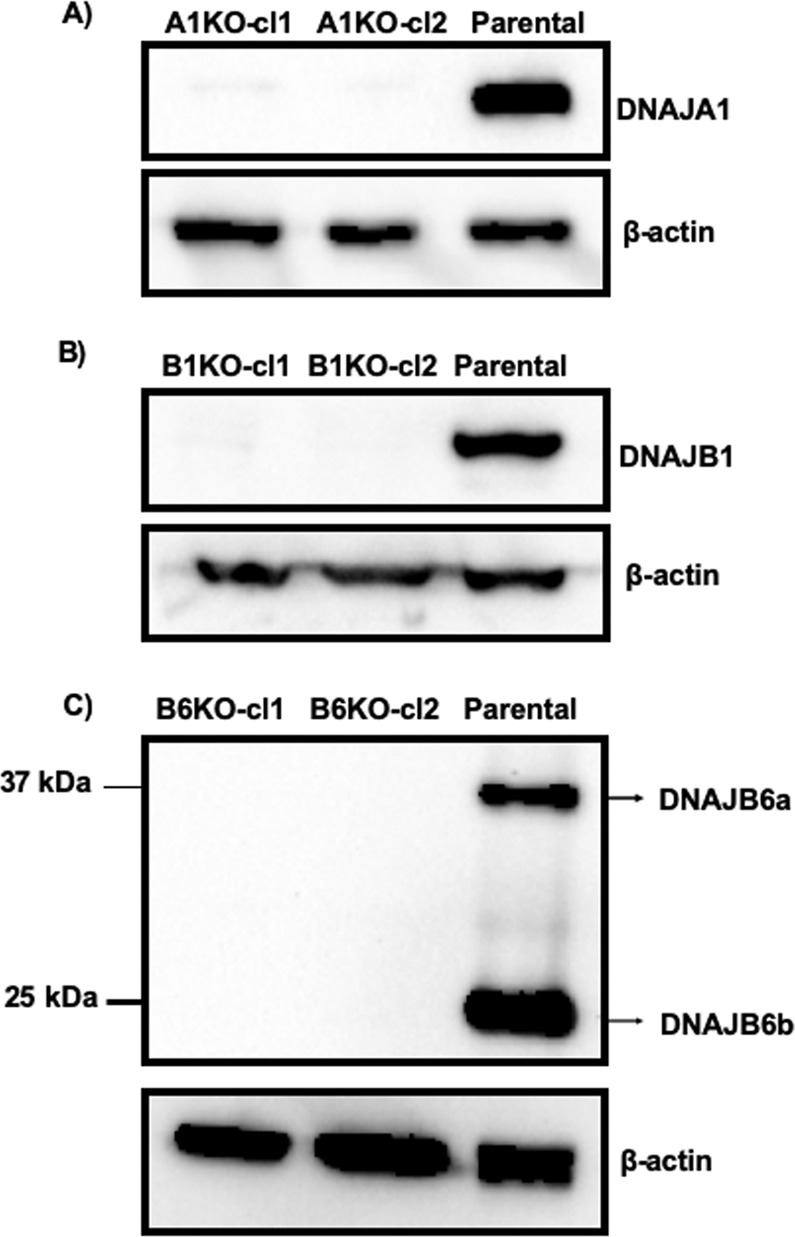
Western blot analysis displaying knock-out of DNAJ genes. KO of DNAJA1(**A**) DNAJB1 (**B**) and DNAJB6 (**C**) were analysed by probing the membranes with anti-DNAJA1, anti-DNAJB1 and anti-DNAJB6 antibodies respectively.

In order to study the influence of DNAJA1, DNAJB1 and DNAJB6 on polyQ aggregation, we generated a stable HEK293 cell line with constitutive expression of a stretch of 74 glutamine residues coupled to huntingtin exon 1 and GFP (polyQ74htt-GFP). Subsequently, we generated KO cell lines for the genes we wanted to study, by creating indel mutations using the CRISPR/CAS9 technology. The potential KO cells were FACS sorted and KO’s of the genes, resulting in loss of expression of the proteins they encode, respectively, were verified by western blotting (Fig. 1a–c).

**Figure 2:**
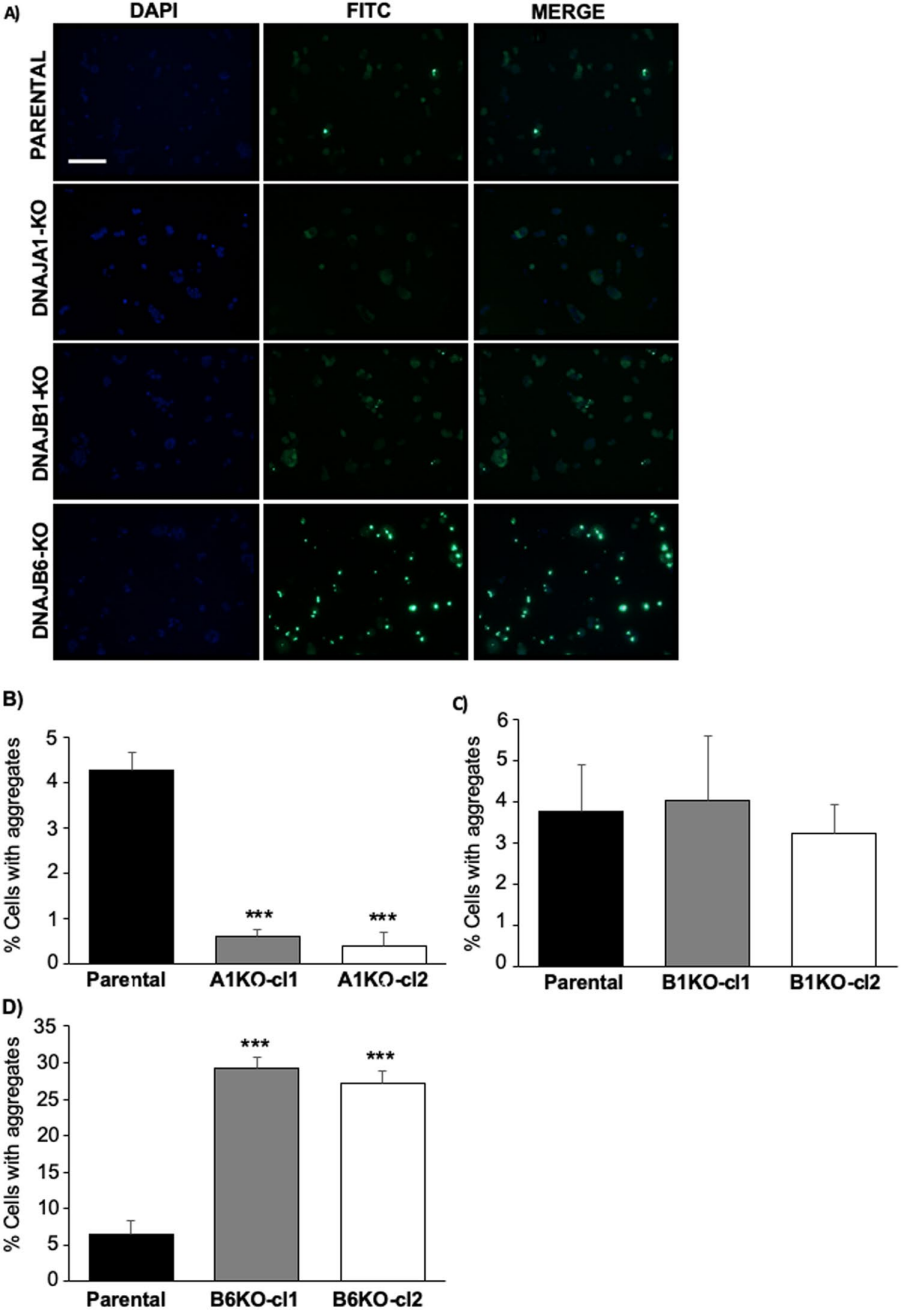
KO of DNAJ genes results in changes in polyQ74htt aggregation. (**A**) Representative fluorescent microscopy images displaying polyQ74htt aggregates and quantification of this in DNAJA1KO **(B**), DNAJB1KO(**C**) and DNAJB6KO (**D**) cells compared to parental polyQ74htt HEK293 control cells, scale bar = 50 µm. ***p < 0.001, analysed by one-way ANOVA. (n = 3).

**Figure 3:**
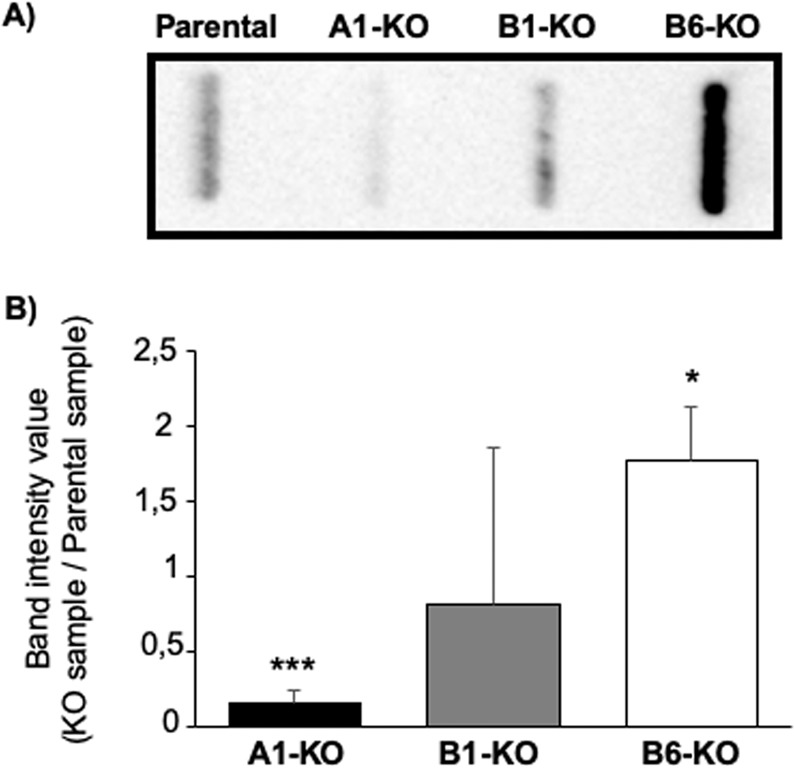
Filter trap assays shows that KO DNAJA1 and DNAJB6 alters polyQ74htt-GFP aggregation. (**A**) Representative image of the assay. (**B**) Data analysis of 5 filter trap assays, the value of the intensity band of each assay is divided by the parental band, in order to obtain the fold change of aggregates of the specific knock out cell lines. Membranes were analysed by probing with anti-GFP antibody. *p < 0.05, ***p < 0.001, analysed by one-way ANOVA. (n = 5).

In order to analyse the effect of these KO’s on polyQ aggregation, we evaluated the frequency of cells with polyQhtt-GFP aggregates (puncta) using fluorescence microscopy (Fig. 2A and Supplemental Fig. 1). Analysis were performed for these KO’s, two independent clones in each case, and compared to the parental control cell line. The results revealed that, whereas DNAJB6 KO resulted in an approximately 5 times increase of aggregates number, KO of DNAJA1, unexpectedly, substantially decreased the amount of polyQhtt-GFP aggregates about 4 times. In the case of DNAJB1 KO, no significant difference in amount of aggregates was found compared to parental cell line (Fig. 2B–D). To verify that the observations seen by fluorescence microscopy were caused by a change in polyQhtt aggregation, we analysed the cell lysates by FTA. In FTA, polyQhtt-GFP aggregates from cell lysates were trapped on onto membranes, which were subsequently stained with anti-GFP antibody. The results confirmed that DNAJA1 KO resulted in a strong decrease in amount of aggregates in polyQ74htt-GFP expressing cells and a significant increase in amount of aggregates was seen in DNAJB6 KO cells (Fig. 3A,B). We observed that the effect of knocking out DNAJB6 had a less pronounced effect, when analysed by FTA assay, than by fluorescence microscopy, but we believe that it might be that a different range of aggregates are detected by FTA, compared to fluorescence microscopy, which could explain these differences. Additional analysis by isolation of NP40 insoluble fractions verified that DNAJA1 KO cells had much less insoluble polyQ74htt than did the parental cell line (Supplemental Fig. 2). However, there was no difference in this analysis when parental and DNAJB6 KO cells were compared (Supplemental Fig. 2).

**Figure 4:**
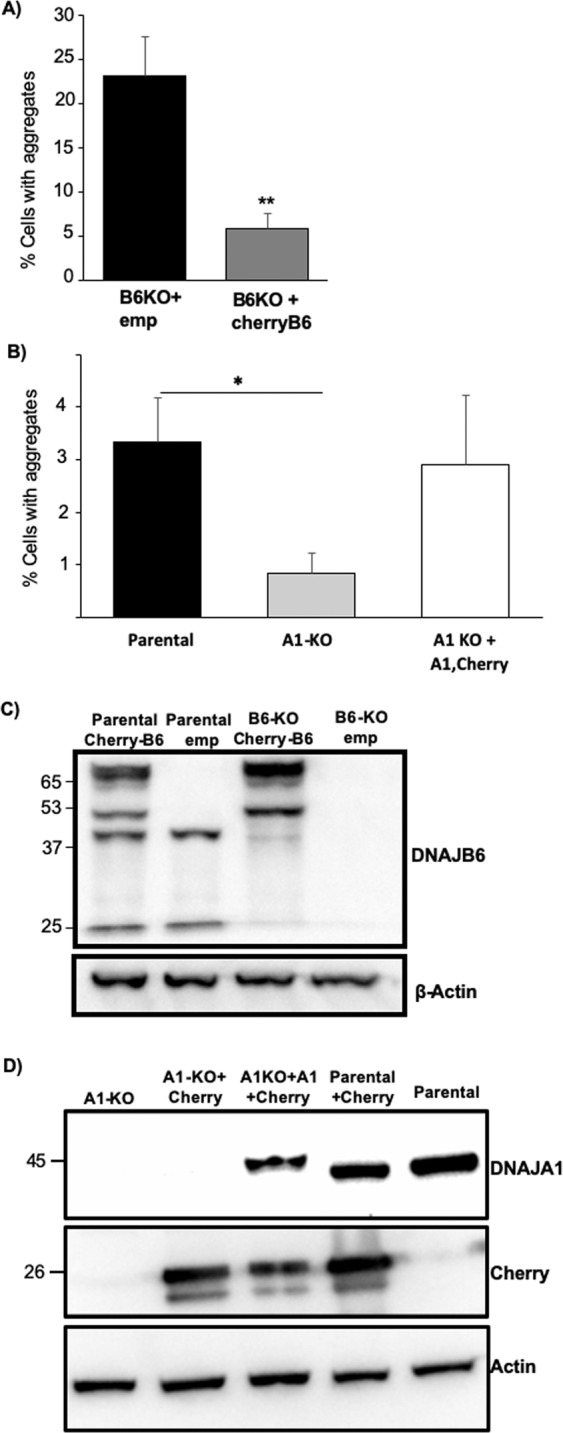
Rescue experiments displaying that restoring expression of the individual proteins in cell with KO of DNAJB6 (**A**) or DNAJA1 (**B**), caused the level of polyQ74htt aggregation to return to the levels of parental control cells. Verification of re-introduction of DNAJB6 (**C**) or DNAJA1 (**D**) into DNAJB6 KO or DNAJA1 KO HEK293 cells respectively. Emp: Empty vector control. *p < 0.05, **p < 0.01, analysed by one-way ANOVA. (n = 3).

To evaluate if the effect of the KO’s was specific to knocking out the genes we wanted to target, we performed rescue experiments to re-introduce either DNAJB6 or DNAJA1 into these KO cells. When we re-introduced DNAJB6 into DNAJB6 KO cells, we observed that the frequency of cells with polyQ74htt aggregates were lowered from 23% to 5%, which is the level of the parental cells, showing that the effect of KO was indeed due to the specific KO of DNAJB6 (Fig. 4A). When we re-introduced DNAJA1 into DNAJA1 KO cells, the cells showed a strong tendency towards elevated frequency of aggregates compared to A1 KO cells and had comparable amount of aggregates compared to the level in the parental cell line (Fig. 4B). The verification of re-introduction of DNAJB6 and DNAJA1, respectively, was verified by WB analysis (Fig. 4C,D).

**Figure 5:**
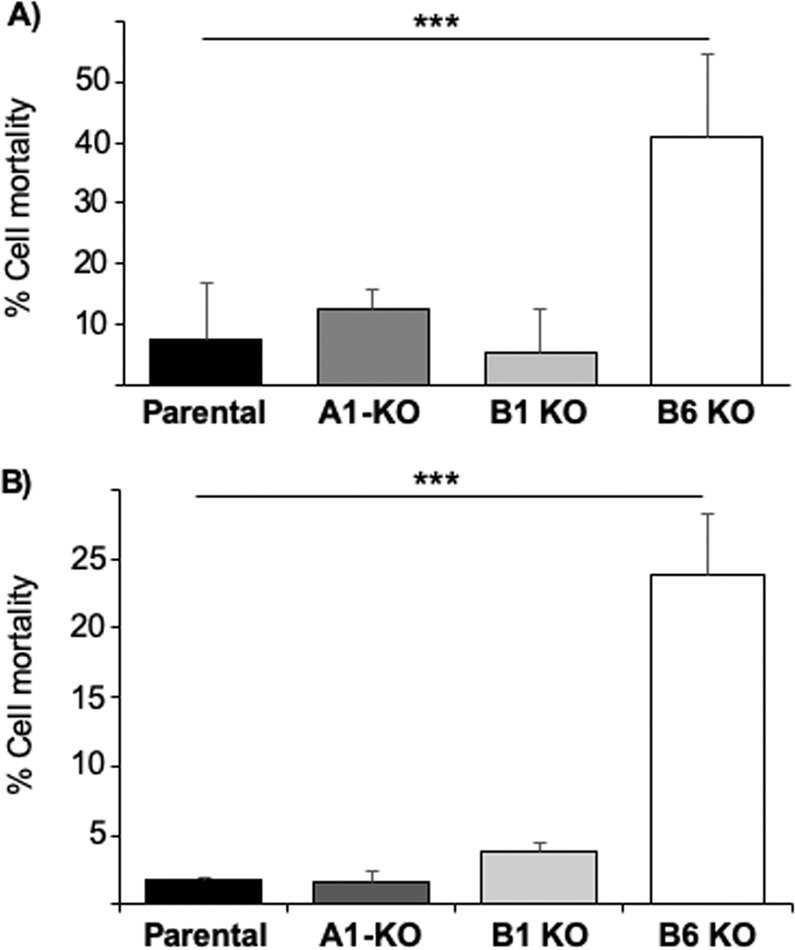
(**A**) Trypan blue exclusion assay displaying difference in cell death in B6-KO compared to control cells. (**B**) Cell death as analysed by flow cytometry using PI uptake assay. ***p < 0.001 as analysed by one-way ANOVA. (n = 3).

It is well known that polyQhtt aggregation can result in cell death of affected cells in disease. In order to test if the observed increased aggregation would result in increased cell death, we analysed this by trypan blue exclusion assay and PI uptake assay, respectively. The results show that there is a link between the large increase in polyQ74htt aggregation seen in the DNAJB6 KO cells and a substantial increase in cell death as well (Fig. 5A,B and Supplemental Fig. 3). These results further confirm that DNAJB6 is important for protection against polyQhtt aggregation and cell death.

## Discussion

Accumulation of misfolded aggregated protein can be a cause of neurodegeneration and consequently disease. DNAJ proteins are co-chaperones that can facilitate protein clearance or promote protein folding, which are mechanisms that can counteract accumulation of protein aggregates. In case of HD, a polyQ expansion at the N-terminus of the huntingtin protein is the cause of this mono-genetic disease. Therefore, we wanted to explore if three DNAJ proteins would modify the aggregation propensity of polyQ74htt. These specific DNAJ proteins, DNAJA1, DNAJB1 and DNAJB6, where chosen as they have previously been shown to modify polyQhtt or other amyloid protein aggregation^15,17,18^. We choose to investigate how knock out of the endogenous levels of three proteins in a polyQ74htt expressing HEK293 cell line would affect the aggregation status of polyQ74htt.

We verified this affect by multiple different methods: We analyzed by amounts of cells that contained GFP positive puncta, by filter trap assay or by analysis of fractions of cells into insoluble and soluble fractions. All of these methods are widely used assays to evaluate polyQhtt aggregation^21–23^. We observed that all of the 3 types of assays confirmed that there was a several fold decrease in amount of aggregates detected in DNAJA1 KO’s compared to parental cells, while there was a dramatic increase in amount of aggregates seen in DNAJB6 KO cells compared to parental cell fluorescence microscopy analysis and filter trap assay but not by analysis of insoluble fractions (Figs. 2, 3 and Supplemental Fig. 2). Whereas these are all valid assays to analyze amount of aggregated polyQhtt we do not expect that the same range of aggregates are detected in all of these assays. Moreover, the fluorescence microscopy analysis enabled analysis of individual cells whereas the filter trap and insoluble fraction analysis only allowed for analysis of the total population of cells. These are reasons why the fold difference between parental and KO cells cannot be expected to be the same between assays. Moreover, several studies by lab of Harm Kampinga have also concluded that DNAJB6 is a major suppressor of polyQ aggregation supporting the results of this study^12,16^.

Several studies have previously investigated the potential of DNAJB6 to prevent amyloid protein aggregation. Specifically, DNAJB6 has been shown to suppress aggregation of polyQhtt when overexpressed in cells^12,24^ and even to delay HD like disease progression when overexpressed in brain of R6/2 mice^16^. In addition, we have shown that KO of DNAJB6 from HEK293 cells causes an increase in alpha-synuclein aggregation, as well as that DNAJB6 can delay alpha-synuclein aggregation *in vitro*^15^, and other *in vitro* studies have suggested that DNAJB6 may even delay aggregation of Abeta42 amyloid protein as well^25^. These results suggest that DNAJB6 may suppress aggregation of multiple amyloid proteins in cells. With regards to DNAJA1 and DNAJB1 less has been reported regarding their potential to alter amyloid protein aggregation but it has been demonstrated that DNAJA1 can modify tau aggregation in cells^18^ and that DNAJB1 can disaggregate alpha-synuclein fibrils *in vitro*^17^.

In this study, we observed increase in polyQ74htt aggregation when we knock out DNAJB6 from HEK293 cells overexpression polyQ74htt (Fig. 2A,D). This is in line with findings by others^12^, but in this study we conclude these findings by knocking out the endogenous levels of DNAJB6 as well as by rescue experiments, rather than relying on overexpression. Whereas DNAJB1 KO did not alter polyQ74htt aggregation, we found that KO of DNAJA1 resulted in a dramatic decrease in amount of polyQhtt aggregates (Fig. 2A,B).

Previously, others have concluded that another DNAJ protein, DNAJB8, is also able to suppress polyQ aggregation^12,24^. However, as DNAJB8 is almost exclusively expressed in testis it is not likely to play a role in HD. With data supporting that DNAJB6 can suppress aggregation of polyQhtt in cell and animal models as well as in cells and *in vitro* of several other amyloid proteins it cannot be excluded that DNAJB6 is a master suppressor of amyloid protein aggregation of multiple amyloid proteins. However, it cannot be ruled out that some of the DNAJ proteins may form a network that prevent amyloid protein aggregation in different cell types and/or cellular compartments. In this context it is worth to note that three DNAJ genes, DNAJB2, DNAJC6 and DNAJC13 have been genetically linked to rare forms of Parkinson’s Disease (PD)^9,11,26^. It remains to be explored if these may affect the aggregation of the PD associated protein alpha-synuclein or if these genetic links are unrelated to this.

It is widely believed that the major function of DNAJ proteins is to serve as co-chaperones of the HSP70 proteins, which are of major importance for protein folding in the cell. Therefore, it is not directly evident how the KO of a DNAJ gene can result in a decrease of polyQhtt aggregation. The effect of DNAJA1 may be caused by serving a dominant negative effect on other DNAJ proteins through heterodimerization or through an entirely different mechanism, which remains to be explored. Furthermore, DNAJA1 and DNAJB6 belong to different subclasses (A and B) of the DNAJ family of proteins^27^(Supplemental Fig. 4), and there may be general structural differences between these DNAJ subclasses that could be of importance for how they can modulate amyloid protein aggregation. In summary, we have demonstrated in this study that the DNAJ proteins are important regulators of polyQhtt aggregation. It is possible that DNAJ proteins may be good targets for future drug designs.

## Supplementary information

figure 1-4.
