# Supplementary material for: Co-chaperones DNAJA1 and DNAJB6 are critical for regulation of polyglutamine aggregation
Source: Sci Rep. 2020 May 18;10:8130. doi: 10.1038/s41598-020-65046-5 (PMC7235262; doi:10.1038/s41598-020-65046-5)
Supplement: Supplementary file 1 — figure 1-4. [file 41598_2020_65046_MOESM1_ESM.pdf]

Supplementary information

Co-chaperones DNAJA1 and DNAJB6 are critical for regulation of polyglutamine aggregation

Claudio Rodríguez-González[1], Shiying Lin[1], Sertan Arkan[1], Christian Hansen[1]

[1]Molecular Neurobiology, Department of Experimental Medical Science, Lund University, BMC B11, 22184, Lund, Sweden.

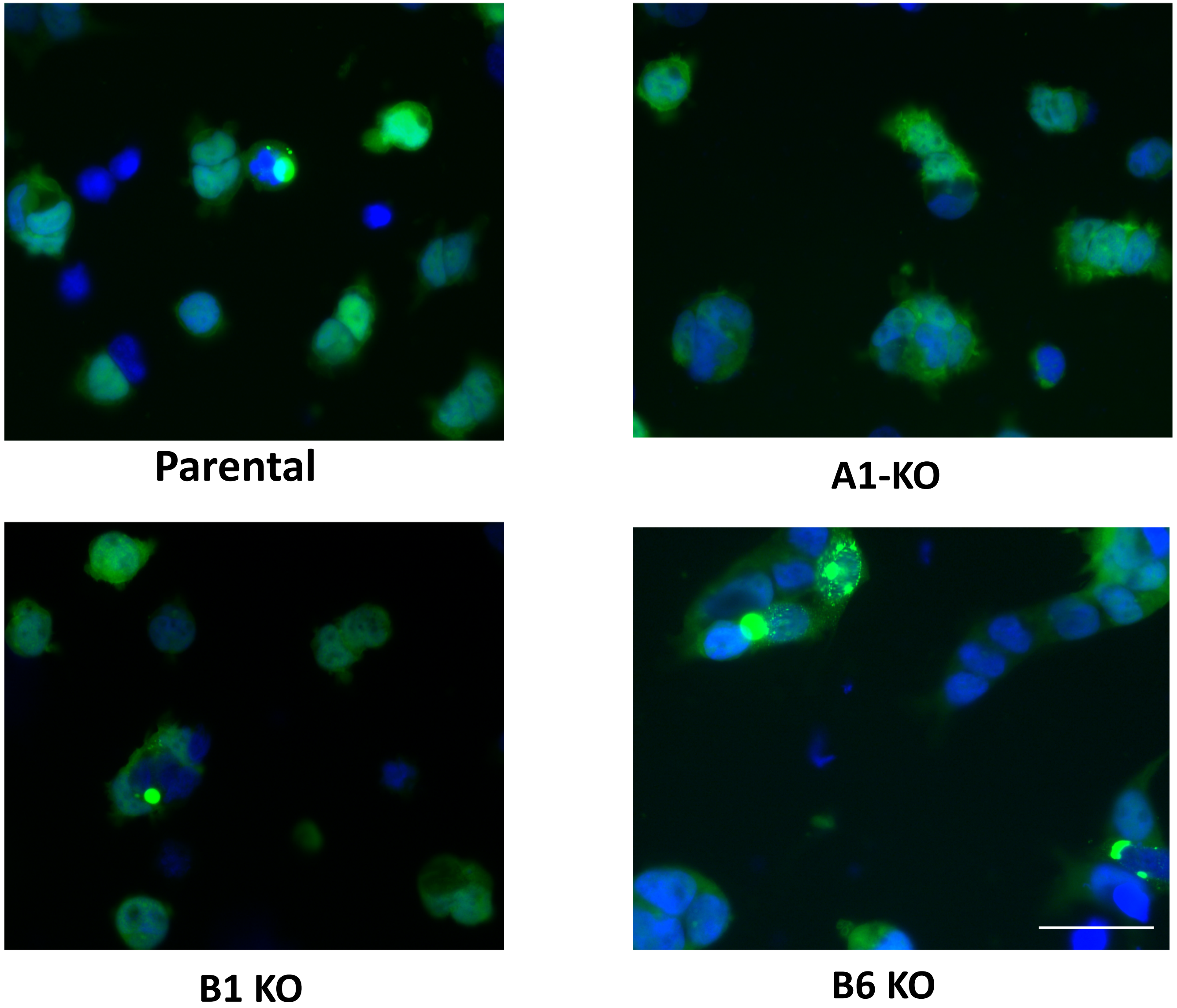

Supplemental figure 1: Representative pictures displaying polyQhtt-GFP puncta in parental and DNAJ KO cell lines using 40x magnification lense of Nikon eclipse 80i microscope. Scalebar: 50 µm

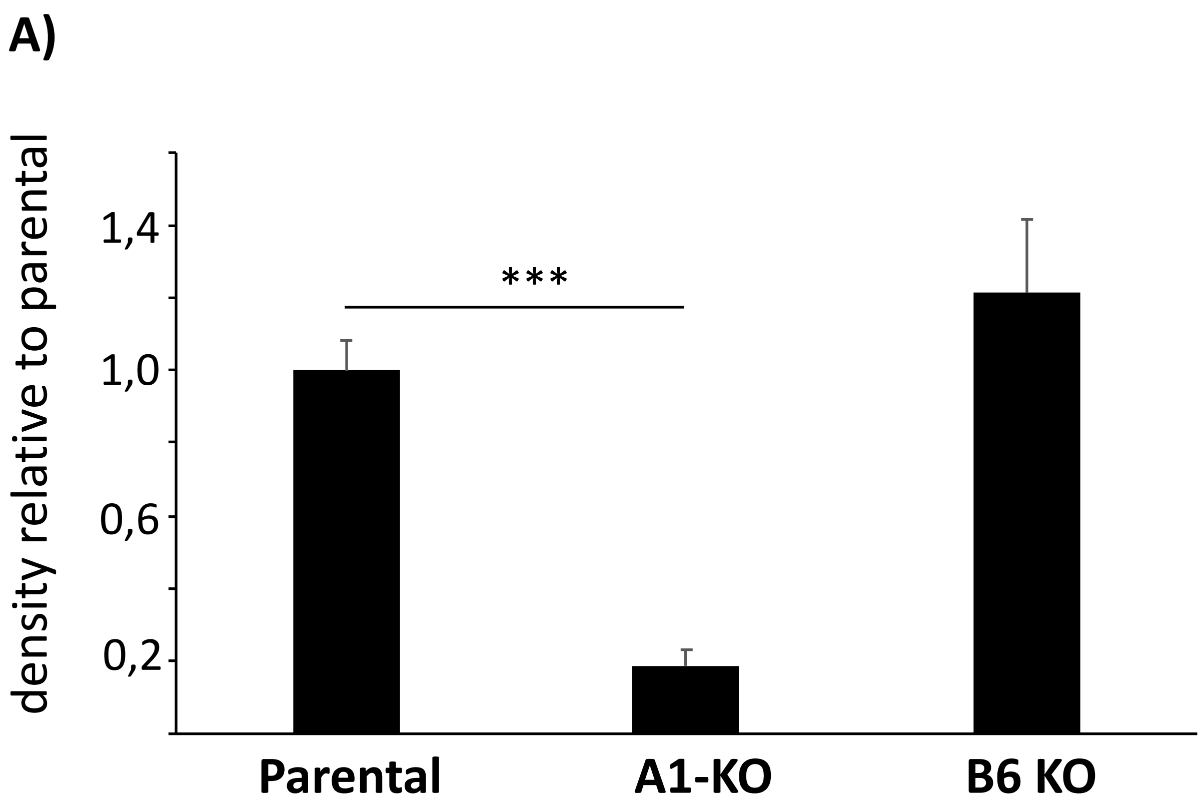

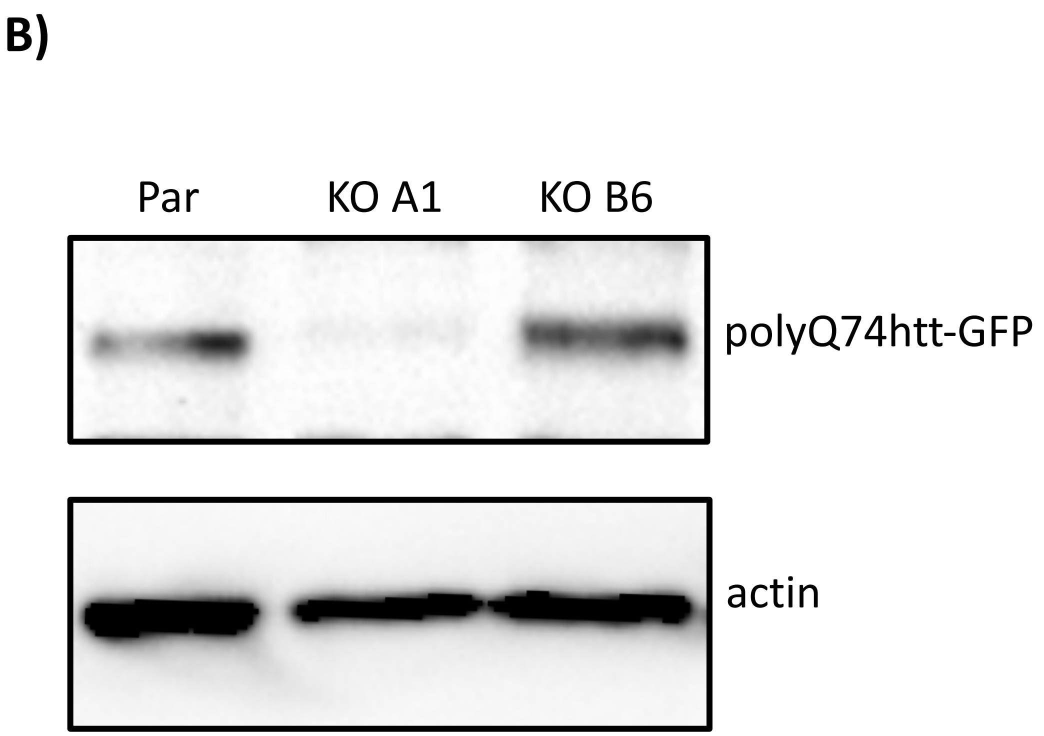

Supplemental figure 2: Analysis of polyQ74 in insoluble fraction of parental of KO cell lines. **A,** Quantification of amount of polyQ74htt-GFP in insoluble fractions of parental or DNAJ KO cells. **B**, representative blot displaying the amount of polyQ74htt-GFP in insoluble fractions of parental or DNAJ KO cells as analysed by staining of blots with mouse anti-GFP and goat anti-mouse HRP (n=11), *** P<0.001 as analyzed by 1-way ANOVA

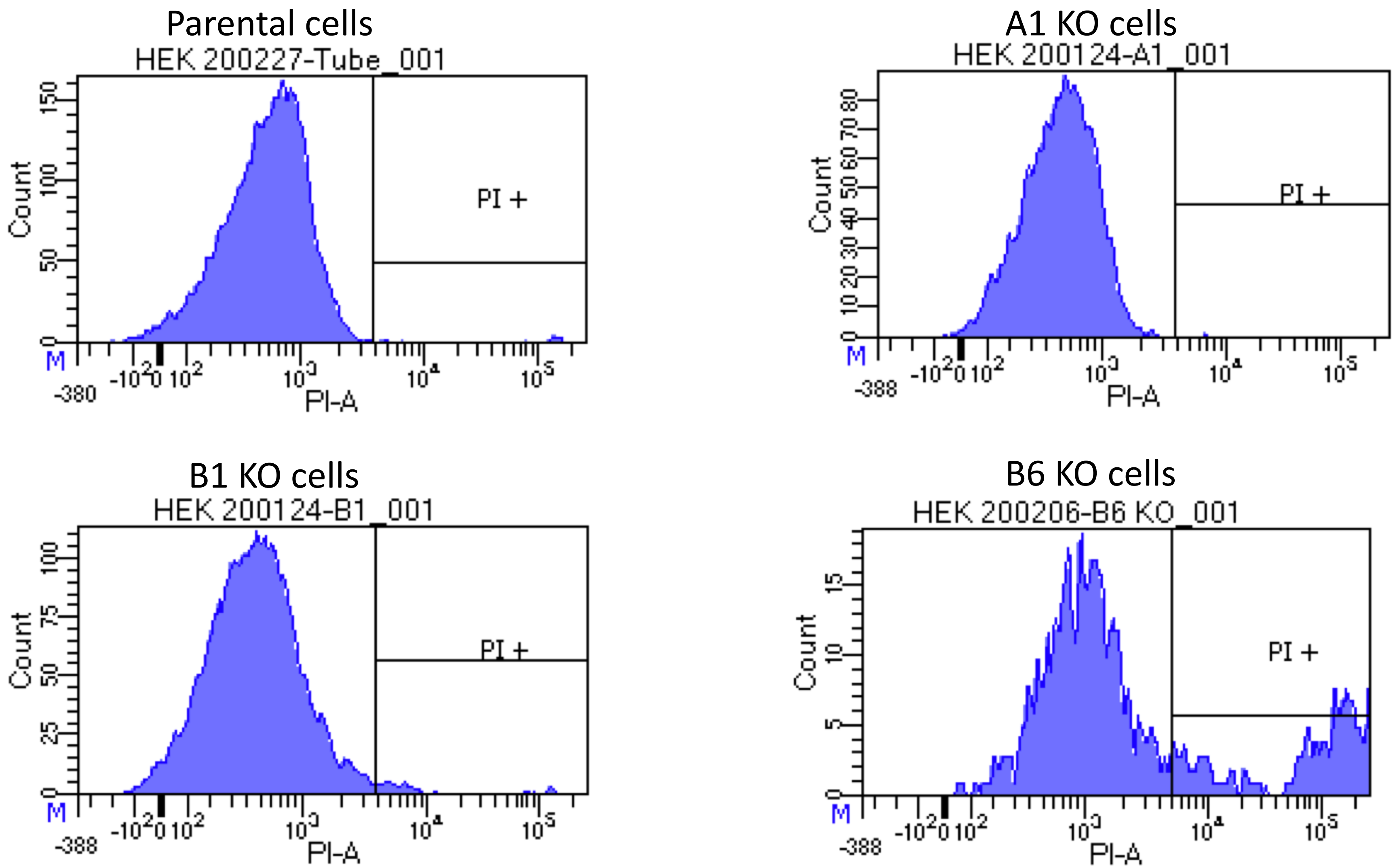

Supplemental figure 3: Propidium iodide uptake based assessment of cell death. Representative pictures from Flow cytometry analysis

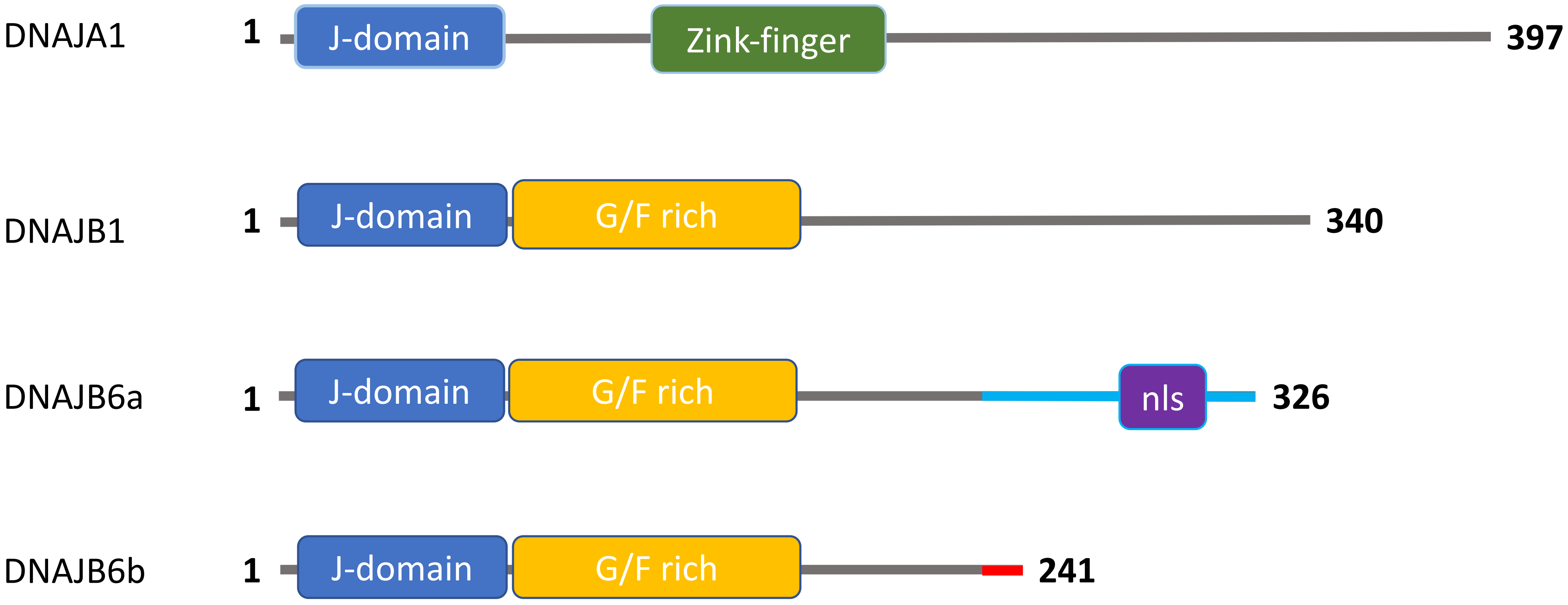

Supplemental figure 4: Overview of domains within DNAJA1, DNAJB1 and DNAJB6, as well as splice form differences between isoform ”a” and ”b” of DNAJB6. The J domain (blue) is common for all 4 proteins, whereas a G/F rich sequence (yellow) is present in DNAJB1, DNAJB6a and DNAJB6b. DNAJA1 carries a putative Zink finger domain (green). The DNAJB6 gene encodes two isoforms (a and b). The longer isoform (a) has a nucleus localization signal at the C-terminus (nls, purple). The 85 aa at the c-terminus (aa 232-326 , light blue) is unique to the a isoform, whereas the 10 aa at the c-terminus of the b-isoform (aa232-241, red) is unique to the b isoform
